# Dynamics of peripheral immune signature identified by multi-omics and its impact on recurrence after radiofrequency ablation of hepatocellular carcinoma

**DOI:** 10.3389/fimmu.2026.1760329

**Published:** 2026-02-20

**Authors:** Kang Li, Wanting Shi, Yongjun Li, Yi Song, Baochen Du, Jinxia Guan, Jianjun Li, Dandan Guo, Tingting Mei, Ang Li, Yonghong Zhang

**Affiliations:** 1 Biomedical Information Center, Beijing You’An Hospital, Capital Medical University, Beijing, China; 2 Beijing Key Laboratory (BZ0373), Beijing You’An Hospital, Capital Medical University, Beijing, China; 3 Interventional Therapy Center for Oncology, Beijing You’An Hospital, Capital Medical University, Beijing, China; 4 Intrnational Medical Services, Beijing Chaoyang Hospital, Capital Medical University, Beijing, China; 5 BioChain (Beijing) Science and Technology, Inc., Beijing, China; 6 Institute of Clinical Medicine, Beijing Friendship Hospital, Capital Medical University, Beijing, China

**Keywords:** hepatocellular carcinoma, integrative analysis, radiofrequency ablation, recurrence prevention, self-developed methylation array, T cell differentiation, transcriptome and methylome

## Abstract

**Background:**

Radiofrequency ablation (RFA) has emerged as a commonly used approach for early-stage hepatocellular carcinoma (HCC) patients. Exploring immunity changes after RFA therapy is helpful for reducing recurrence.

**Methods:**

In this study, we enrolled 12 patients with HCC with their 47 blood samples, including before and after complete RFA therapy. We performed an integrative analysis of the transcriptome and methylome, investigated using a novel self-developed methylation array (HYGEIA panel). Core analyses included differential analyses of both transcriptome and methylome, DIABLO-based multi-omics integration, gene set enrichment analysis, and time-series gene clustering with visualization.

**Results:**

Our study elucidated the complex effect of the location of CpG site methylation on their corresponding gene transcription; 58.44% of CpG sites were located in the promoter (≤1 kb) region and mainly negatively correlated with gene expression. RFA treatment in HCC patients activated antigen processing and presentation and Th1 and Th2 cell differentiation signaling pathways. The anti-tumor immune responses induced by RFA therapy persisted for less than 9 months in recurrent patients. Meanwhile, the ability of T-cell differentiation in HCC patients was a potential factor to prevent recurrence.

**Conclusion:**

These findings elucidated the dynamic peripheral immune remodeling post-RFA and identified host T-cell fitness as a key determinant of recurrence, providing a rationale for combining RFA with immunotherapy to prolong protective immune responses.

## Introduction

Radiofrequency ablation (RFA) induces local tumor necrosis while preserving as much normal liver tissue as possible and has emerged as a commonly employed radical approach for early-stage hepatocellular carcinoma (HCC) patients. Meanwhile, RFA can modulate the immune system and anti-tumor immune activation (1). Proliferation of tumor-specific T cells induced by RFA treatment was associated with reduced recurrence of patients with HCC (2). RFA induced a functional activation of myeloid dendritic cells, which was associated with increased serum levels of tumor necrosis factor-α (TNF-α) and IL-1beta in HCC patients (3) and changed levels of Th1/Th2 cytokines (4). RFA promoted T-cell infiltration in some animal models (5), significantly increased GPC3-specific cytotoxic T lymphocytes (CTLs) compared to surgical resection (6), induced the number of antigen-loaded dendritic cells (DCs) in lymph nodes (LNs) (7), and enhanced CD4(+) T-cell immune responses (8).

However, a high recurrence rate was also observed after RFA treatment, as with all the other HCC therapeutic modalities (9). Methyltransferase-1 played an immunosuppressive role in the accumulation of polymorphonuclear myeloid-derived suppressor cells in the tumor microenvironment after RFA (9). Fortunately, immunotherapy following RFA treatment is a promising solution to recurrence. The interim analysis of the IMbrave050 study indicated that adjuvant treatment with atezolizumab combined with bevacizumab following ablation or curative resection could reduce the risk of disease recurrence in HCC patients with a high risk of postoperative recurrence (10). In our preliminary exploratory study, we also found that postoperative adjuvant programmed cell death protein 1 (PD-1) inhibitor therapy for HCC patients at high risk of recurrence after complete ablation significantly delayed disease recurrence. The 1-year recurrence-free survival (RFS) rate was 73.3% in the PD-1 inhibitor group versus 46.7% in the control group (11). Anti-CTLA-4 blockade enhanced tumor-specific T-cell response induced by RFA (12), and anti-PD-1 blockade boosted RFA promotion of T-cell infiltration by overcoming PD-L1 overexpression. Thus, the complexity of RFA-induced immunity and its impact on recurrence still need further exploration.

According to observations at the level of clones based on single-cell T-cell receptor sequence data, peripheral and intratumoural clones were significantly correlated (13). Peripheral blood mononuclear cells (PBMCs) are critical components of the host immune system and a direct reflection of the global immune status. Collecting PBMCs is more practical than tissue in monitoring immune changes caused by RFA treatment, as the latter is easy to obtain in surgical resection studies. The contradictory role of immunosuppression and activation induced by RFA in PBMCs of recurrent HCC patients implied that deep immune mechanisms were complex and that the integrity of the experimental control was essential (14). Therefore, we sought to identify peripheral immune changes before and after RFA with over 14 months of follow-up and whether recurrence occurred or not in genome-wide DNA methylation and gene expression. Our systematic multi-omics analysis showed that RFA induced anti-tumor immune responses that persisted for less than 9 months in recurrent patients, and the potential ability of T-cell differentiation in patients with HCC was also critical for early recurrence prevention.

## Materials and methods

### Patients

In this study, 12 HCC patients with their 47 blood samples were included before and after complete RFA therapy in You’An Hospital from October 2017 to December 2019. The diagnosis of HCC was preferably histologically confirmed. In cases when tumor biopsy results were unavailable, diagnosis was established by contrast-enhanced CT or MRI. Treatment in each patient was performed by an interventional radiologist with >5 years of experience, guided by the National Comprehensive Cancer Network (NCCN) and Chinese HCC treatment guidelines. Complete ablation is defined as the presence of an ablative margin of at least 5 mm around the entire tumor, no enhancement in the arterial phase, and no defect in the portal phase on enhanced CT scan. Patients underwent follow-up every 1 month in the first 3 months post-RFA and every 3 months thereafter. The longest follow-up time was 14 months. The definition of early recurrence was the relapse of HCC within 1 year after complete ablation. Patient characteristics are shown in **Table 1** and **Supplementary Table 1**.

**Table 1:** Clinical and pathological characteristics of 12 patients with HCC with their 47 longitudinal samples.

Patient ID	Age	Gender	Tumor stage (BCLC)	Child–Pugh score	Recurrence status	Pre-ablation (1w–3w before)	Post1m (1m–2m)	Post6m (4m–7m)	Post9m (8m–10m)	Post12m (12m–14m)
Pat1	44	M	A	A	NR	**+**	**+**		**+#**	**+**
Pat3	58	M	A	A	NR	**+#**	**+**	**+#**		
Pat5	33	M	A	A	NR	**+#**	**+**	**+**	**+#**	**+**
Pat6	63	M	0	A	NR	**+#**		**+**	**+#**	
Pat9	65	M	0	A	NR	**+#**	**+**	**+**	**+**	**+#**
Pat2	75	M	A	A	R	**+#**		**+**	**+**	**+#**
Pat4	53	M	A	A	R	**+#**	**+**	**+**	**+#**	
Pat7	62	M	A	B	R	**+#**	**+**	**+**		
Pat8	67	F	A	A	R	**+#**	**+**	**+**	**+**	**+#**
Pat10	59	M	A	B	R	**+**	**+**	**+#**		
Pat11	47	M	A	A	R	**+#**	**+**	**+**		**+**
Pat12	51	M	A	A	R	**+**	**+**	**+**		**+**

Gender: M, male; F, female. Recurrence status: R, recurrence in a year; NR, non-recurrence in a year.

m, month; w, week; BCLC, Barcelona Clinic Liver Cancer staging system; PBMC, peripheral blood mononuclear cell; RFA, radiofrequency ablation; +, PBMC samples used for RNA-Seq; #, PBMC samples used for DNA methylation; _, paired samples before and after RFA treatment.

The analyses included in this study were performed in accordance with the Declaration of Helsinki and were approved by the Ethics Committee of the Beijing You’An Hospital (Jing You Ke Lun Zi [2022]055# in Chinese). Written informed consent from the patients for the research use of data was obtained before the investigation.

### DNA methylation array and raw data processing analysis

The fresh blood samples were collected in Ethylenediaminetetraacetic acid (EDTA) tubes, and then genomic DNA was extracted and bisulfite-converted (ZYMO Research, Irvine, CA, USA) for the DNA methylation assay. The DNA methylation test panel named HYGEIA was constructed by BioChain (Beijing) Science & Technology Inc. (Beijing, China). It contained 5,117,910 CpG sites on a single-stranded DNA chain, and the detailed information is shown in **Supplementary Table 2**. The single-chain library was constructed by BioChain and was used for the methylation sequencing of target interval capture.

First, the sequencing quality of the raw data was analyzed using the Fastp software (15) to remove low-quality bases/reads and obtain clean reads. The Bismark alignment software (16) was used to align clean reads with the reference genome (GRCh38). After deduplication, “Bismark_sthylation_detractor” was used to extract CpG sites and count the number of reads. The methylation level of a single CG site = number of reads of the C base/the total number of reads; the methylation level of a gene = the number of reads covering C bases within the gene/the total number of reads within the gene.

### RNA sequencing and raw data processing analysis

Total RNA was extracted using TRIzol (Invitrogen, Life Technologies, Carlsbad, USA) and assessed using Agilent 2100 Bioanalyzer (Agilent Technologies, Santa Clara, CA, USA) and Qubit Fluorometer (Invitrogen). Total RNA samples that met the following requirements were used in subsequent experiments: RNA integrity number (RIN) > 7.0 and a 28S:18S ratio > 1.8. RNA-sequencing (RNA-Seq) libraries were generated and sequenced by CapitalBio Technology (Beijing, China). The final libraries were quantified using the KAPA Library Quantification kit (KAPA Biosystems, Cape Town, South Africa) and Agilent 2100 Bioanalyzer. After quantitative reverse transcription–polymerase chain reaction (RT-qPCR) validation, libraries were subjected to paired-end sequencing with paired-end 150-bp read length on an Illumina NovaSeq sequencer (Illumina, Inc., San Diego, CA).

The human genome version of hg38 was used as a reference. The sequencing quality was assessed using FastQC (v0.11.5) (17), and then low-quality data were filtered using NGSQC (v2.3.3) (18). The clean reads were then aligned to the reference genome using HISAT2 (v2.1.0) (19) with default parameters. The processed reads from each sample were aligned using HISAT2 against the reference genome. The gene expression analyses were performed using StringTie (v1.3.3b) (20).

### Differential analysis and correlation analysis

Four paired samples (pre-treatment and post-treatment) of non-recurrent patients and three paired samples of recurrent patients were applied to differential expression/methylation analysis.

Differentially expressed genes were identified using the DESeq2 package in RNA-Seq count data. Differentially methylated CpG sites were identified using the Limma package in methylation rate data.

The calculation of the correlation coefficient of the methylation of each CpG site with its corresponding gene expression was performed using Spearman’s correlation in a nested for-loop. The gene regions were categorized into nine groups: promoter (2–3 kb), promoter (1–2 kb), promoter (≤1 kb), 5′UTR, exon, intron, distal intergenic region, 3′UTR, and downstream.

### mixOmics data integration and gene set enrichment analysis

DIABLO mixOmics (version 6.30.0) (21) with sparse partial least-squares discriminant analysis (sPLS-DA) was applied for the integrative analysis of transcriptome with methylome. Gene expression levels were quantified as Transcripts Per Million (TPM) and log2-transformed [log_2_(TPM + 1)]. To account for potential inter-sample technical variation, quantile normalization was further performed across all samples prior to integrative analysis. Similarly, quantile normalization was applied across all samples to mitigate batch effects. A total of nine pre-treatment and nine post-treatment samples with transcriptome and methylome datasets were taken for integration. DIABLO was used to identify highly correlated gene signatures across two omics datasets. To identify key immune processes that were differentially activated or suppressed between experimental groups, gene set enrichment analysis (GSEA) was conducted using the gseKEGG functions implemented in the “ClusterProfiler” package. The normalized enrichment score (NES) > 1 and adjusted *p*-value < 0.05 in GSEA results were defined as significant.

### Clustering and visualization of time-series gene analysis

The ClusterGVis package was applied to cluster and visualize the time-series gene expression data of the transcriptome. TPM values were log_2_-transformed by dividing the number of reads mapped to a transcript by the transcript length. TPM normalization of RNA-Seq data was used for the ClusterGVis package. The elbow method was used to define five as a suitable cluster value based on the total within sum of squares value. The *mfuzz* method could be chosen to cluster data and detect genes with a consensus trend. Gene symbol was converted to gene ID using the database “org.hs.eg.db”, and enrichment analysis was performed. The “enrichCluster” function was used to enrich immune-related pathways in the “BP” type with *p*-value < 0.05.

### Analysis of T-cell differentiation signatures and validation of prognostic value

To evaluate the association between T-cell differentiation signatures and the risk of recurrence after radiofrequency ablation, the following analyses were performed. First, four T-cell differentiation-related pathway gene sets were obtained from the C5 collection of the MSigDB database (http://www.gsea-msigdb.org/gsea/msigdb): regulation of T-cell differentiation in the thymus, regulation of gamma-delta T-cell differentiation, positive regulation of CD8-positive alpha-beta T-cell differentiation, and positive regulation of CD4-positive alpha-beta T-cell differentiation. The corresponding signature scores for each patient’s pre-treatment sample were calculated using the gene set variation analysis (GSVA) algorithm. Then, stratified analysis was performed to compare RFS outcomes between the high-score and low-score groups. For survival analyses, the Kaplan–Meier curves were generated, and log-rank tests were used to derive *p*-values. Hazard ratios (HRs) and 95% confidence intervals (CIs) were estimated using Firth’s penalized likelihood (to address complete separation in subgroups with no events). To validate the independent prognostic value of T-cell differentiation signatures, univariate and multivariate Cox proportional hazards regression analyses were conducted. The multivariate models incorporated clinical confounders, including age, gender, Barcelona Clinic Liver Cancer (BCLC) stage, and the Child–Pugh score. For external validation, The Cancer Genome Atlas Liver Hepatocellular Carcinoma (TCGA-LIHC) cohort (n = 371) was analyzed using the same T-cell differentiation gene sets, with multivariate adjustment for clinical factors (sex, age, tumor stage, and grade).

## Results

### Correlation analysis of methylation of CpG sites with their corresponding gene expression

**Figure 1:**
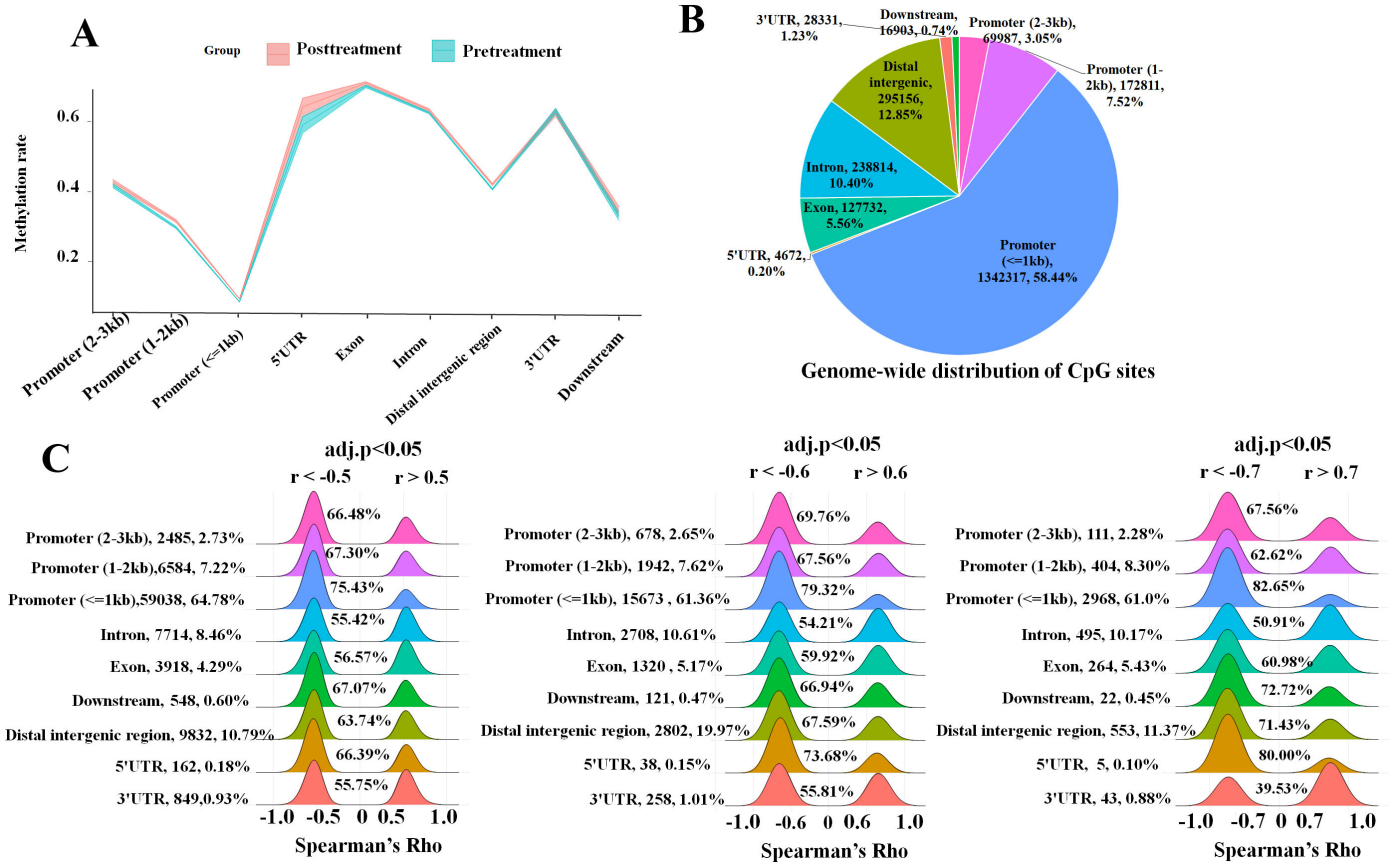
Correlation between methylation of CpG sites and their corresponding gene expression. **(A)** DNA methylation levels of promoter (2–3 kb), promoter (1–2 kb), promoter (≤1 kb), 5′UTR, exon, intron, distal intergenic region, 3′UTR, and downstream. **(B)** Pie charts illustrate the genome-wide distribution of CpG sites in nine gene regions. **(C)** Ridge plot shows Spearman’s rho distribution in the nine gene regions. Left, |r| > 0.5; middle, |r| > 0.6; right, |r| > 0.7. In each panel, the left percentage indicates the proportion of each item, and the right percentage indicates the proportion of negative correlation coefficients.

**Figure 2:**
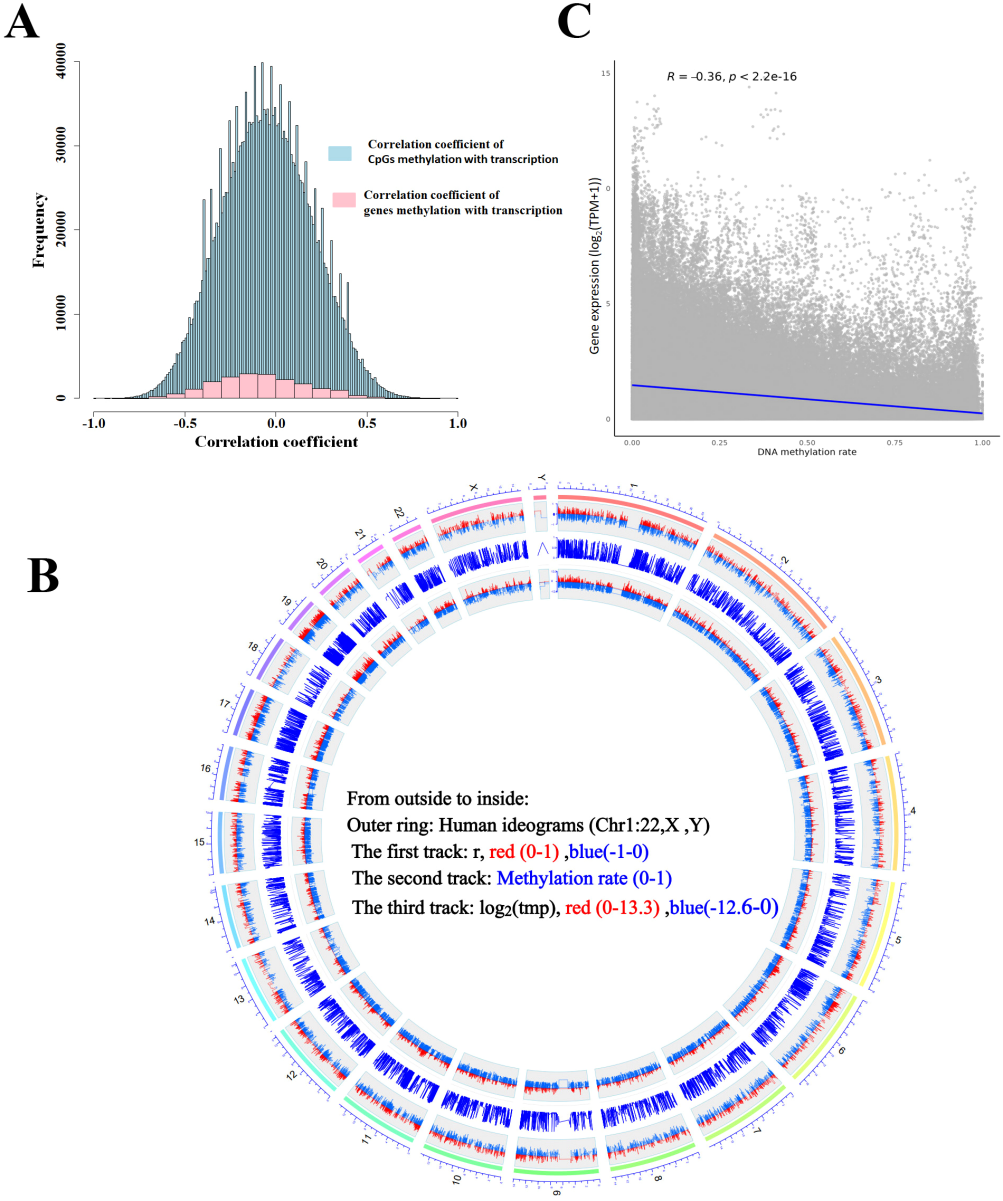
The correlation of gene methylation with its expression. **(A)** Histogram plot shows the frequency of the correlation coefficient of CpGs or gene methylation levels with transcription. **(B)** Scatter plot shows a correlation between methylation and transcription of 18,626 genes. **(C)** Circular plot of genome-wide correlation between DNA methylation and gene transcription. The outer ring represents human chromosome ideograms. The first track represents differential correlation between methylated CpG sites and transcription of corresponding genes (|r| > 0.5, adjusted *p*-value < 0.05). The second track represents methylation ratio level of CpG sites. The third track represents genes transcription level.

After methylation HYGEIA panel and RNA-Seq assays, a total of 18 samples were used in this section (**Table 1**). Over 2.2 million CpG sites were measured, with the majority located in promoter regions, particularly within 1 kb of transcription start sites, where methylation levels were the lowest (**Figures 1A, B**). All detected CpG sites were mapped to over 18,000 genes, and most genes contained more than 30 measurable CpG sites. Tens of thousands of CpG sites whose methylation significantly correlated with expression (adj. *p* < 0.05, |r| > 0.5) were identified, with approximately 75% residing in promoters. Most promoter-proximal CpGs showed negative correlation with gene expression, and this inverse relationship became more pronounced closer to the transcription start site and with stronger correlation coefficients (**Figure 1C**). At the gene level, average CpG methylation intensity demonstrated a significant moderate negative correlation with expression across all samples (Spearman’s R = −0.36, *p*-value < 0.001) (**Figure 2**, **Supplementary Table 3**). In summary, these results indicated that the correlation between DNA methylation and mRNA expression was complex and not simply a negative correlation.

### DIABLO integration analysis of transcription and methylation between pre-treatment and post-treatment samples of RFA

**Figure 3:**
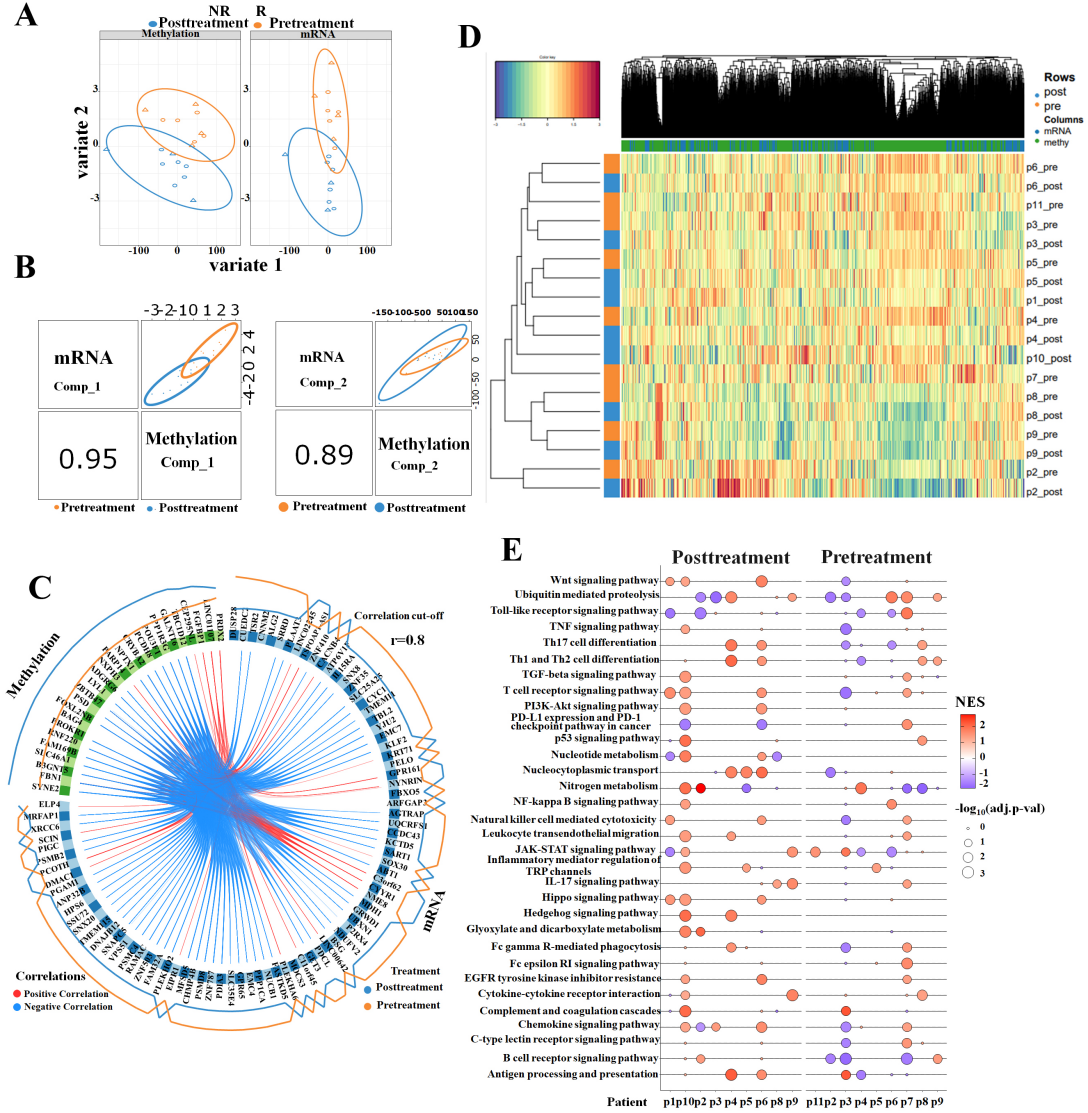
Sparse partial least-squares discriminant analysis (sPLS-DA) between pre-treatment and post-treatment. **(A)** The contribution of methylome and transcriptome to sPLS-DA final model: methylome showed better separation capability than transcriptome data in the first two components. **(B)** Scatterplot of two datasets (upper diagonal plot) and Pearson’s correlation between each dataset (lower diagonal plot) in the first component (left) and the second component (right). **(C)** Circos plot of correlations (cut-off value of r = 0.8) based on the sPLS-DA results using the transcriptome (blue) and methylome (green) data of the first two components. **(D)** Unsupervised analysis between mRNA scores and methylation scores is displayed by a heatmap. The color of each gene or methylation site was based on its contribution scores to the first two components. **(E)** Genes whose correlations between mRNA and methylation were above 0.7 underwent GSEA. GSEA evaluation of the median rank of these gene signatures in pre- and post-treatment PBMC samples. An adjusted *p*-value less than 0.05 was considered significant. NES, normalized enrichment score, NES > 0, activated; NES < 0, inhibited. GSEA, gene set enrichment analysis; PBMC, peripheral blood mononuclear cell.

The sPLS-DA was applied to identify a subset of variables that could explain the variability between nine pre-treatment and nine post-treatment samples. It was demonstrated that the methylome data had a higher variance than the transcriptome data for the first two components (**Figure 3A**). A global overview of the correlation at the component level is displayed in **Figure 3B**. It revealed the correlation between the methylation data and transcription data, resulting in a correlation of 0.95 and 0.89 for the first and second components, respectively. All the correlations between pre-treatment and post-treatment samples assessed in multi-omics were shown in a circos plot (**Figure 3C**) with a cut-off level of 0.8. The result revealed a number of strong negative correlations. Furthermore, unsupervised analysis shown by heatmap clustering (**Figure 3D**) demonstrated that the highly correlated genes (>0.7) between multi-omics mostly distinguished pre-treatment and post-treatment samples. These gene signatures underwent GSEA to evaluate the median rank in all PBMC samples. The results showed that RFA treatment enhanced immune activity, leading to increased NES of antigen processing and presentation, Th1 and Th2 cell differentiation, and T- and B-cell receptor signaling pathway and reduced NES of PD-L1 expression and PD-1 checkpoint pathway in cancer (**Figure 3E**). In conclusion, the mixOmics method indicated that the gene signatures with strong correlation between multi-omics play important roles in exploring immune profiles of ablation treatment.

### Differential analysis of gene expression and DNA methylation between recurrent and non-recurrent patients

**Figure 4:**
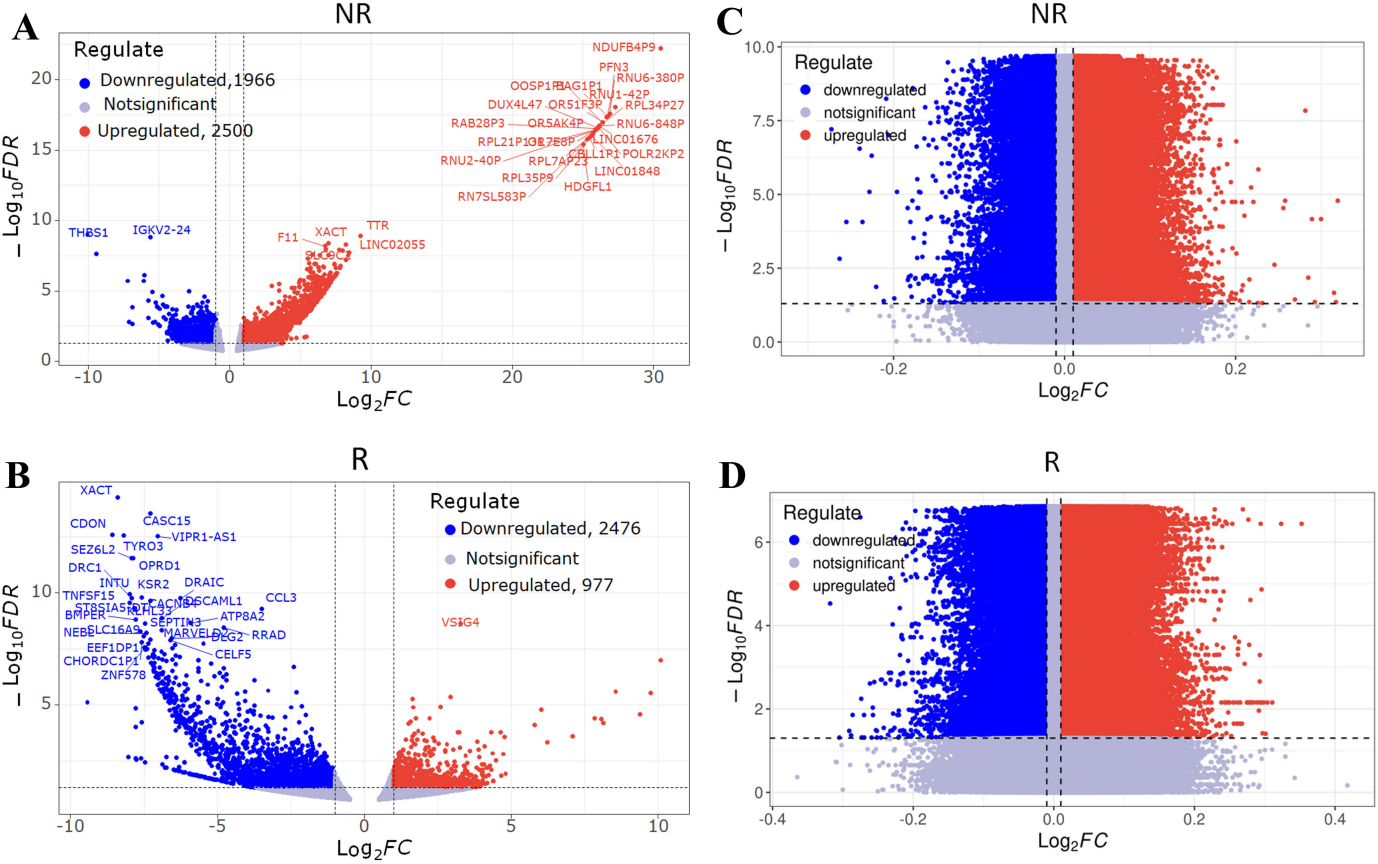
Differentially expressed genes and differentially methylated CpG sites in post-treatment samples compared to pre-treatment of RFA therapy in HCC patients. Volcano plot shows differentially expressed (DE) genes in non-recurrent patients **(A)** and recurrent patients **(B)**. Thresholds: |FC| = 1.0, FDR *p*-value = 0.05. Upregulated genes and downregulated genes are marked with red points and blue points, respectively. Volcano plot shows differentially methylated (DM) CpG sites in non-recurrent patients **(C)** and recurrent patients **(D)**. Thresholds: |FC| = 0.01, FDR *p*-value = 0.05. Upregulated CpG sites and downregulated CpG sites are marked with red points and blue points, respectively. RFA, radiofrequency ablation; HCC, hepatocellular carcinoma; FC, fold change.

We analyzed RNA-sequencing count data from 14 paired samples (four non-recurrent and three recurrent patients) using the DESeq2 package. With an False Discovery Rate (FDR) threshold of < 0.05, we identified a set of differentially expressed (DE) genes in both patient groups: non-recurrent patients showed more upregulated than downregulated DE genes, while recurrent patients exhibited the opposite trend (**Figures 4A, B**). For DNA methylation analysis, we used the Limma package to assess methylation rates in the same paired samples. Using the same threshold, we detected numerous differentially methylated (DM) CpG sites in both cohorts, with upregulated DM CpG sites significantly outnumbering downregulated sites in both non-recurrent and recurrent patients (**Figures 4C, D**). To ensure the robustness of the results, we performed a sensitivity analysis (**Supplementary Table 4**). Although the number of identified differential features varied across different thresholds, the non-recurrent group had more upregulated than downregulated genes, while the recurrent group was dominated by downregulated genes, remaining consistent across all tested levels.

**Figure 5:**
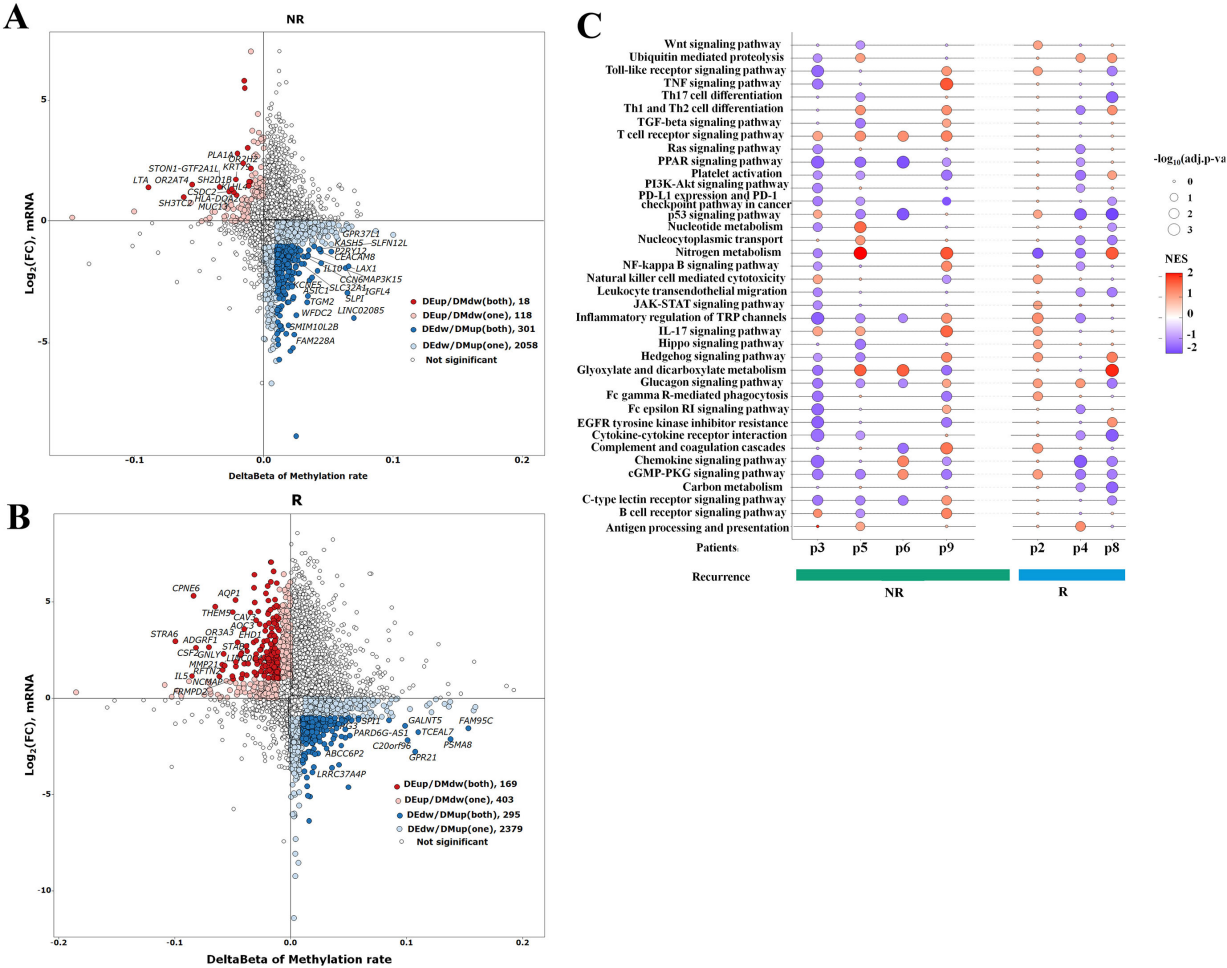
Integrated analysis of transcriptome and methylome of ablation therapy in HCC. Four-quadrant diagram showing genes both differentially expressed and differentially methylated in non-recurrent patients **(A)** and recurrent patients **(B)**. Y axis represents log_2_ (fold change) of mRNAs, and X axis represents the delta beta of methylation rate. X axis (|delta beta| > 0.01) and Y axis (|log_2_ (fold change)>1) were thresholds. DE, differentially expressed; DM, differentially methylated. Red points show DE up and/or DM down, and blue points show DE down and/or DM up. **(C)** Genes that were DE/DM in both or only one comparison are indicated. Genes that were DE and/or DM underwent GSEA. GSEA evaluation of the median rank of these gene signatures in non-recurrent patients and recurrent patients. An adjusted *p*-value less than 0.05 was considered significant. NES, normalized enrichment score, NES > 0, activated; NES < 0, inhibited. GSEA, gene set enrichment analysis.

To analyze how RFA impacts HCC patients with different prognoses, we compared DE and DM genes between the non-recurrent and recurrent cohorts. In non-recurrent patients, RFA treatment suppressed the expression of a subset of genes (characterized by downregulated expression and upregulated methylation) while enhancing the expression of another gene subset (upregulated expression and downregulated methylation) (**Figure 5A**; **Supplementary Table 5**). A similar pattern of gene expression regulation was observed in recurrent patients, with RFA leading to both the downregulation and upregulation of distinct gene sets (**Figure 5B**; **Supplementary Table 6**).

The Kyoto Encyclopedia of Genes and Genomes (KEGG) pathway enrichment analysis of these DE and DM genes revealed the key functional differences between the two groups: antigen processing and presentation, as well as Th1/Th2 cell differentiation signaling pathways, were activated in non-recurrent patients post-RFA and partially in recurrent patients. Additionally, compared to recurrent patients, non-recurrent patients showed inhibition of the PD-L1 expression, PD-1 checkpoint, and TNF signaling pathways (**Figure 5C**).

### The time-series differential expression analysis and pathway enrichment in non-recurrent and recurrent patients

**Figure 6:**
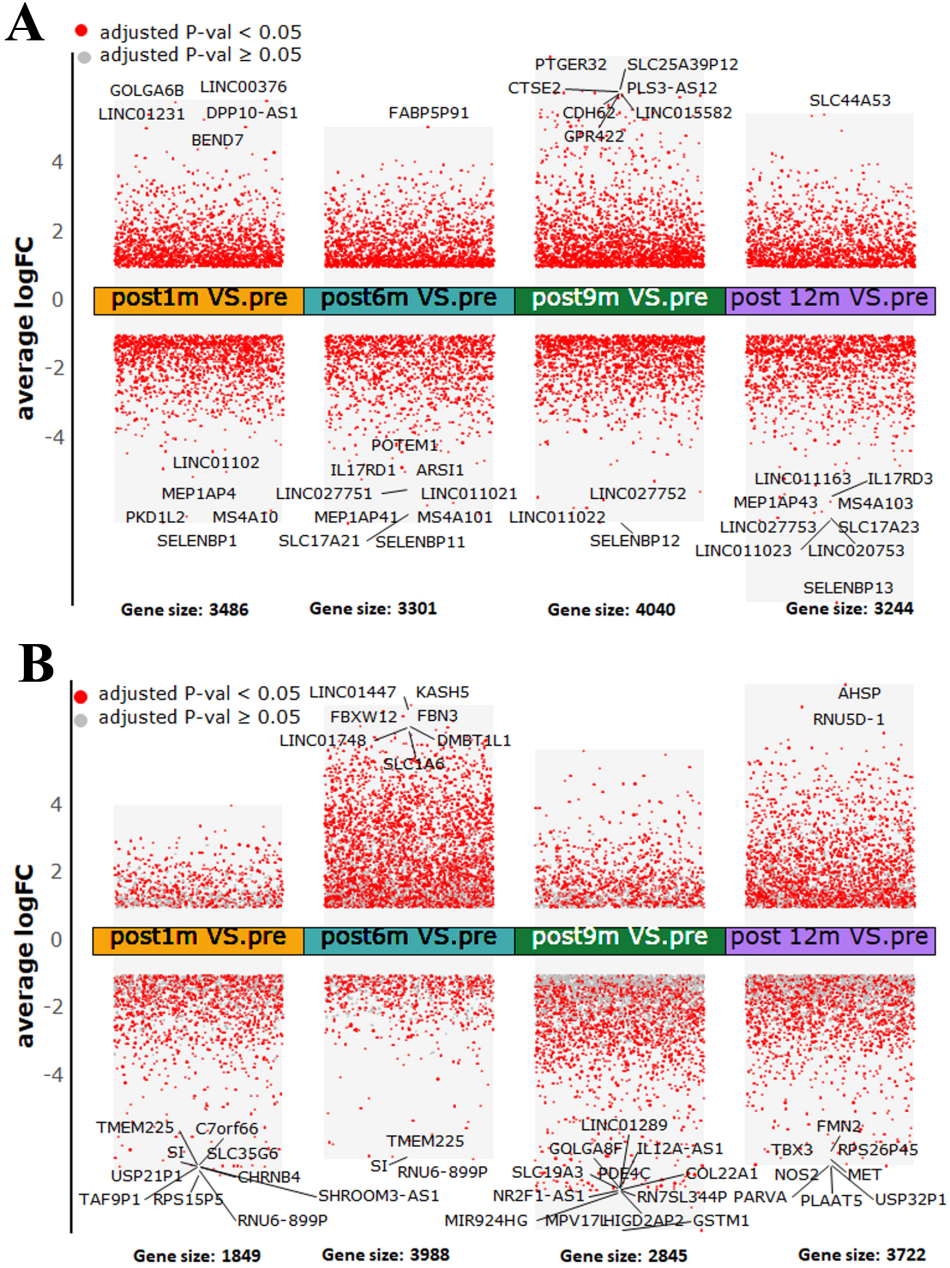
Differentially expressed genes across all four post-treatment periods compared to pre-treatment period in **(A)** non-recurrent patients and **(B)** recurrent patients. An adjusted *p*-value < 0.05 with |log_2_ fold change| > 1 is indicated in red, while an adjusted *p*-value ≥ 0.05 with |log_2_ fold change| > 1 is indicated in gray.

We identified the DE genes of five periods, including pre-treatment and post-treatment at 1, 6, 9, and 12 months in HCC patients who underwent complete ablation therapy. We performed differential gene expression analysis between four post-treatment periods and the pre-treatment period. There were 269 upregulated and 319 downregulated genes in the intersection of differential genes in each time period of post-treatment compared with pre-treatment in the non-recurrent patient group. The selenium binding protein 1 (SELENBP1) was the most significantly downregulated in post-treatment samples of non-recurrent patients (**Figure 6A**; **Supplementary Figure 1**). Meanwhile, we identified 29 upregulated and 140 downregulated genes in the intersection of differential genes in each time period of post-treatment compared with pre-treatment in the recurrent patient group (**Figure 6B**; **Supplementary Figure 1**).

**Figure 7:**
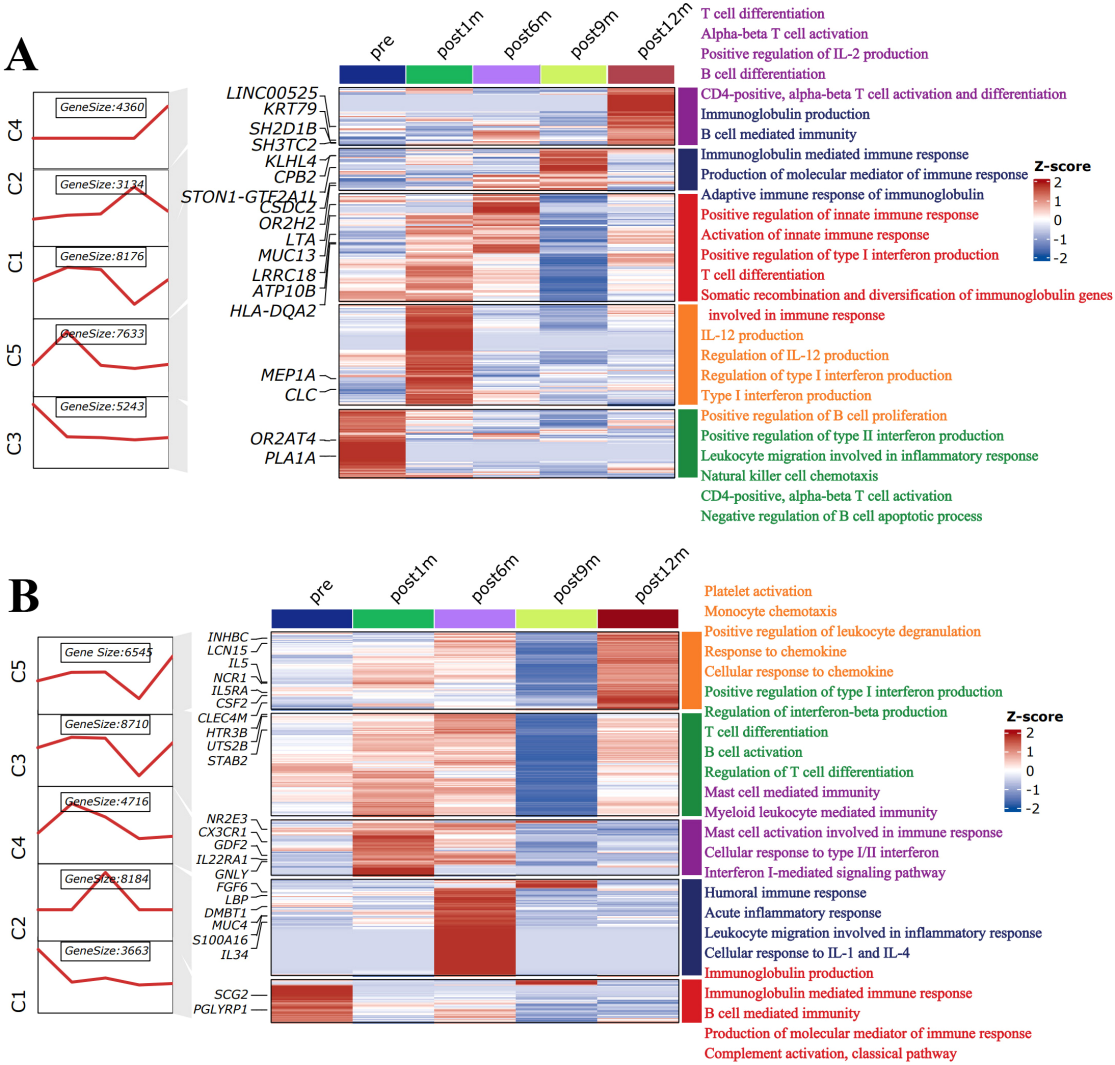
Heatmaps of time-course RNA-Seq expression analysis of pre- and post-ablation treatment states in **(A)** non-recurrent patients and **(B)** recurrent patients. Left line plot represents cluster analysis of gene expression patterns based on Mfuzz. Middle heatmap represents highly expressed gene signatures in pre- and post-ablation treatment states. Right list shows top five enriched immune-related pathways in each cluster with corresponding color. The value for each gene is row-scaled Z-score of gene expression.

To comprehensively investigate the global temporal patterns of the immune-related impact of RFA, we prepared all genes for cluster analysis in different prognoses using the ClusterGVis package. We clustered those genes into five groups (C1–C5) according to their expression patterns in non-recurrent and recurrent patients. We defined the highly expressed genes in five periods using a line map (**Supplementary Figure 2**). Using the specific marker genes, we also performed gene ontology (GO) pathway enrichment analysis for each period. Genes specifically expressed in pre-treatment samples in non-recurrent patients (C3) (**Figure 7A**; **Supplementary Table 7**) enriched more T-cell immune-related pathways, such as T-cell differentiation and T-helper cell differentiation, than the recurrent group (C1) (**Figure 7B**; **Supplementary Table 8**). Ablation treatment induced and enhanced many types of immune responses 6 months in non-recurrent patients (C1 and C5) and recurrent patients (C2, C3, and C4). Nine months after treatment, the adaptive immune system exhibited sustained activation in non-recurrent patients (C2); however, no such immune pathways were enriched in recurrent patients at this time point. At post-treatment 12 months, more types of immune pathways were still activated in non-recurrent patients (C4) compared to recurrent patients (C5). In summary, the RFA therapy in HCC could improve various anti-tumor immune responses persisting for more than 9 months, which would be critical for recurrence prevention. In the meantime, the greater ability of T-cell differentiation in HCC patients was also one of the key factors determining whether recurrence occurred.

### Prognostic value of T-cell differentiation signatures

**Figure 8:**
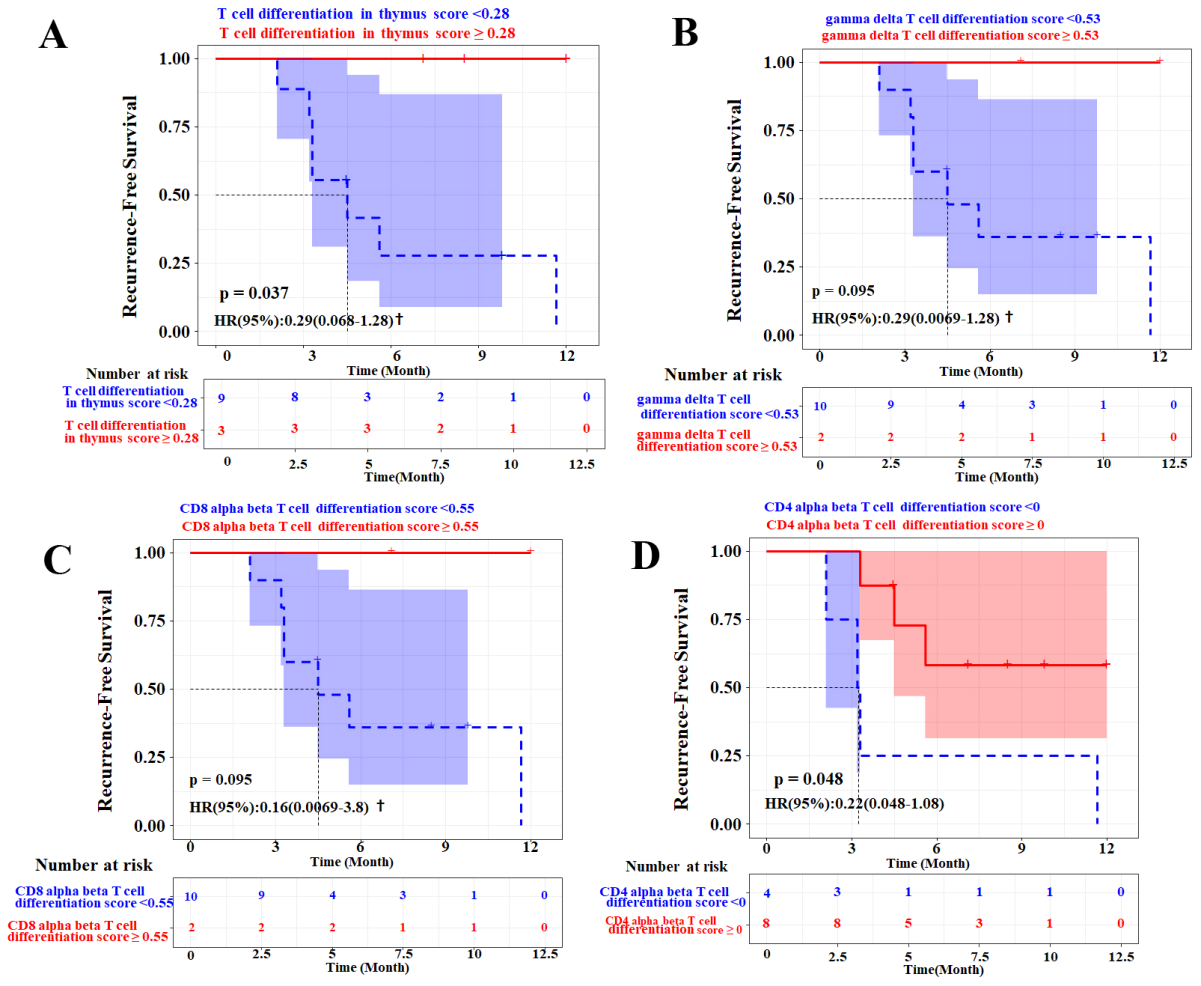
Prognostic value of T-cell differentiation-related gene signatures in pre-ablation treatment samples (n = 12). Kaplan–Meier analysis of recurrence-free survival (RFS) stratified by expression levels of three distinct T-cell differentiation signatures: **(A)** GOBP regulation of T-cell differentiation in thymus, **(B)** GOBP regulation of gamma delta T-cell differentiation, **(C)** GOBP positive regulation of CD8-positive alpha-beta T-cell differentiation, and **(D)** GOBP positive regulation of CD4-positive alpha-beta T-cell differentiation. † denotes hazard ratios (HRs) and 95% confidence intervals estimated using Firth’s penalized likelihood. *p*-Values were derived from log-rank tests. GOBP, gene ontology biological process.

The univariable Cox regression analysis (**Figure 4**) showed that the T-cell differentiation signature score was significantly associated with RFS [*p* < 0.05 for gene ontology biological process (GOBP) regulation of T-cell differentiation in the thymus and GOBP positive regulation of CD4 alpha-beta T-cell differentiation]. The Kaplan–Meier analyses of pre-ablation treatment samples (n = 12) demonstrated that high expression of three distinct T-cell differentiation signatures correlated with improved RFS (**Figures 8A–D**). To further validate whether the T-cell differentiation signature was independently associated with recurrence, we performed multivariable Cox proportional hazards regression analyses (**Supplementary Figure 3**). After adjusting for age, gender, BCLC stage, and the Child–Pugh score, the four T-cell differentiation signatures did not reach statistical significance in the multivariable model. We acknowledged that statistical significance attenuated after multivariable adjustment, likely reflecting the small cohort size with limited events that reduced statistical power, and complete separation requiring Firth’s penalized likelihood correction, which yielded more conservative estimates. However, effect directions remained consistent, and the CD4 alpha-beta differentiation signature showed a particularly strong effect size (HR = 0.10, *p* = 0.060) approaching significance.

**Figure 9:**
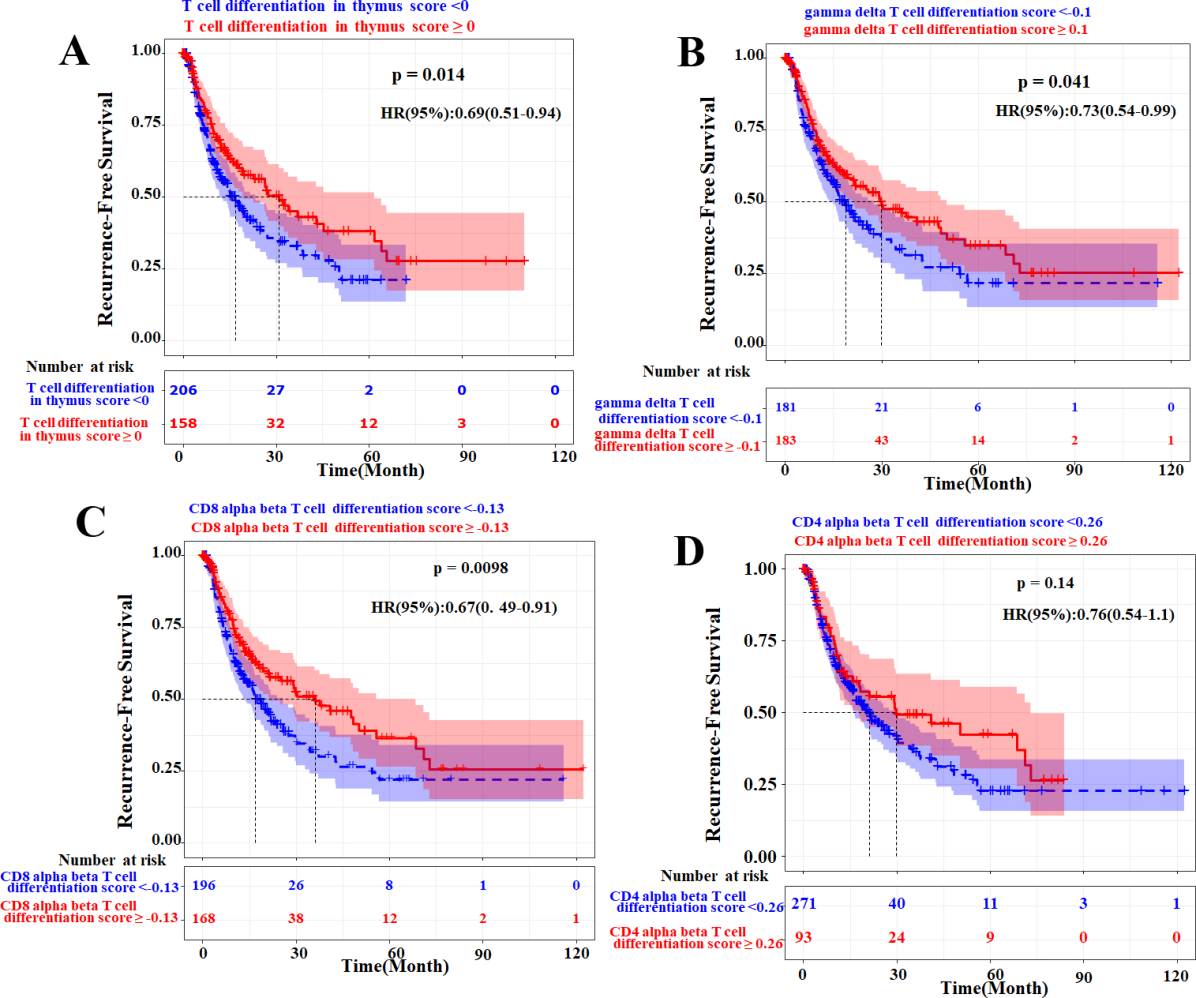
Prognostic value of T-cell differentiation-related gene signatures in hepatocellular carcinoma (TCGA-LIHC cohort, n = 371). Kaplan–Meier analysis of recurrence-free survival (RFS) stratified by expression levels of three distinct T-cell differentiation signatures: **(A)** GOBP regulation of T-cell differentiation in thymus, **(B)** GOBP regulation of gamma delta T-cell differentiation, **(C)** GOBP positive regulation of CD8-positive alpha-beta T-cell differentiation, and **(D)** GOBP positive regulation of CD4-positive alpha-beta T-cell differentiation. Hazard ratios (HRs) and 95% confidence intervals were calculated using Cox proportional hazards model. *p*-Values were derived from log-rank tests. GOBP, gene ontology biological process.

**Figure 10:**
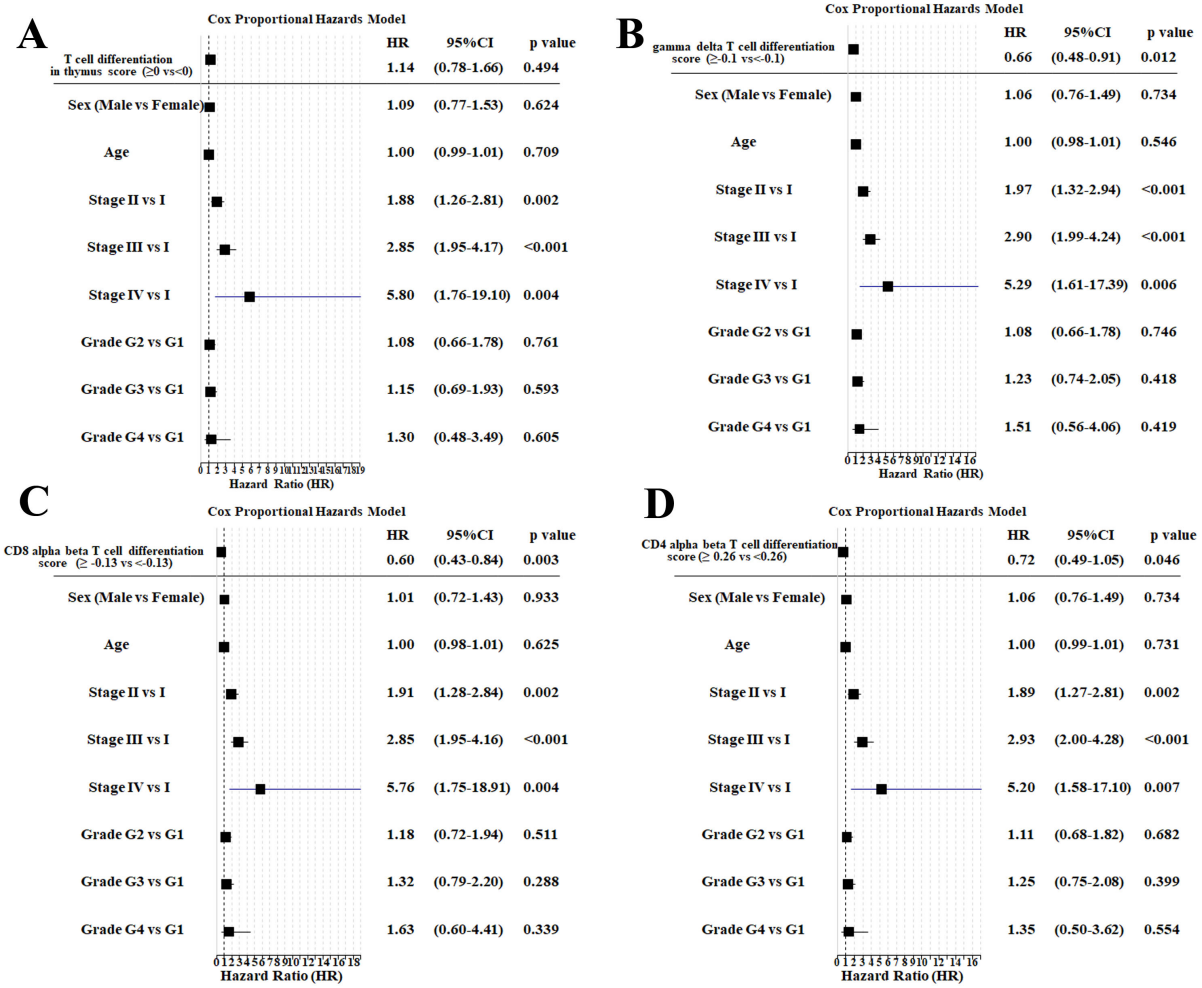
Multivariable Cox regression analysis of T-cell differentiation signatures with HCC recurrence-free survival in TCGA-LIHC (n = 371). Forest plots displaying hazard ratios (HRs) and 95% confidence intervals for **(A)** GOBP regulation of T-cell differentiation in thymus, **(B)** GOBP regulation of gamma delta T-cell differentiation, **(C)** GOBP positive regulation of CD8-positive alpha-beta T-cell differentiation, and **(D)** GOBP positive regulation of CD4-positive alpha-beta T-cell differentiation. HR < 1 indicates protective effect. The vertical red line denotes HR = 1 (no effect). Stage: according to tumor node metastasis classification system. Grade: histologic grade according to the Edmondson–Steiner grading system. GOBP, gene ontology biological process; HCC, hepatocellular carcinoma.

To externally validate the role of host T-cell fitness in predicting recurrence risk, we analyzed the four T-cell differentiation signatures in the TCGA-LIHC cohort (n = 371) via stratified survival analysis (**Figure 9**) and multivariate Cox regression (**Figure 10**). After multivariable adjustment for clinical confounders (sex, age, tumor stage, and grade), the gamma-delta T-cell, CD8 alpha-beta T-cell, and CD4 alpha-beta T-cell differentiation signatures maintained independent prognostic significance, confirming the robustness of these findings. Notably, while the primary cohort used PBMCs and the TCGA-LIHC cohort used tissue, requiring different cutoff values, the trends remained consistent.

## Discussion

Peripheral immune responses after RFA treatment in HCC patients are not completely understood. We selected circulating immune cell (PBMC) samples before and after RFA treatment in non-recurrent patients and recurrent patients to evaluate the potential mechanisms. We applied an integrative analysis of the DNA methylome and transcriptome to explore the effect of RFA treatment on HCC patients.

DNA methylation variability was highly dependent on tissue types and genomic context (22–24). The immune system-related cells/tissues (CD4^+^ T cells, CD8^+^ T cells, and the thymus) showed unique signatures of methylation variability compared to gastrointestinal tissues (24). Meanwhile, the changes in PBMC methylation were affected by the occurrence and progression of HCC (25) and other cancers (26). The interaction between DNA methylation and gene expression in HCC PBMCs had not been reported. Previous studies have shown that the correlation between DNA methylation and gene expression was complex and not simply a negative correlation (27). In the present study, the CpG methylation assay panel provided an opportunity to investigate the correlation between CpG methylation and gene expression. We calculated the correlation coefficient of each CpG site with the mapped genes’ transcription level and accurately assessed this correlation. Overall, the correlation coefficients of all CpG sites presented a normal distribution; the number of negative correlation coefficients and that of positive correlation were roughly the same. As the correlation enhanced, the proportion of negative correlation coefficients of CpG sites in exons, distal intergenic regions, and downstream regions increased, especially in promoter regions and 5′UTR; the situation in 3′UTR and intron was the opposite. These results also supported the viewpoint that the location of CpG had an influence on the effect of methylation on gene transcription (28).

Hänsler et al. reported that RFA treatment increased T-cell amounts and enhanced CD4^+^ and CD8^+^ lymphocyte activity (fivefold increase) in PBMCs of HCC patients (29). Meanwhile, only one out of six HCC patients was found to be locally recurrent within the mean follow-up period of 6 months (29). RFA could enhance the antigen-presenting capacity of PBMCs in recurrent HCC patients (14) and non-recurrent HCC patients (30). However, in another study, the population of CD8^+^ effector and memory T cells significantly decreased in PBMCs of 22 recurrent HCC patients after RFA (14). These interesting results indicated that RFA induced distinct immune effects in different prognostic populations. The ability of T-cell activation and differentiation in HCC patients appeared to be more important for the prognosis of RFA treatment.

Prokhnevska et al. proposed a two-step activation model to explain how CD8^+^ T cells respond to cancer (31) (1). In human tumor-draining lymph nodes (TDLNs), tumor-specific CD8^+^ T cells proliferated, lacking the expression of effector molecules, and this stem-like state (TCF1^+^ CD8^+^ T cells) had been existing for years (31, 32) (2). The effector state of CD8^+^ T cells (TCF1^−^TIM3^+^ terminally differentiated CD8 T cells) required both TCR signaling and co-stimulation in the tumor to acquire effector programming, and this differentiation took several days after their initial activation. The importance of stem-like CD8^+^ T cells was demonstrated in cancer T cell-based immunotherapy, including adoptive cell transfer and checkpoint inhibitor (33–35). Co-stimulation from antigen-presenting cells and signal 3 cytokines (type I IFNs and IL-12) promoted tumor-specific CD8^+^ T-cell differentiation.

Our study evaluated the predictive value of T-cell differentiation signatures for recurrence risk in HCC patients following RFA. In our internal cohort, these signatures showed a consistent protective trend with RFS. Although statistical significance was attenuated after multivariate adjustment, likely due to the limited cohort size (n = 12), the direction of effect remained robust, particularly for the CD4-positive alpha-beta T-cell differentiation signature. This finding was validated in the independent TCGA-LIHC cohort, where multiple T-cell differentiation signatures retained independent prognostic significance after adjusting for clinical covariates. Notably, similar observations have been reported in other tumor types, where CXCR3-mediated T-cell differentiation in renal cell carcinoma was associated with advanced stage and significantly shorter RFS, highlighting its potential as a prognostic biomarker beyond traditional tumor node metastasis staging (36). This cross-cohort and cross-cancer consistency confirms that host T-cell fitness is an intrinsic determinant of HCC prognosis, not merely a surrogate of disease stage. Mechanistically, we found that pre-existing T-cell differentiation capacity, which was enriched only in non-recurrent patients prior to RFA, may serve as a protective baseline. RFA enhanced T-cell differentiation and promoted tumor-antigen release and tumor-specific T-cell activation in all patients (37, 38). However, a key difference emerged in the duration of this response: RFA-induced T-cell differentiation was sustained for no more than 9 months in recurrent patients, whereas its continuous activation appeared crucial for long-term recurrence prevention in the non-recurrent group. This aligns with clinical evidence that combining anti-PD-1 adjuvant therapy with RFA significantly prolongs RFS in high-risk patients (10, 11), potentially by supporting sustained CD8^+^ T-cell effector differentiation and activation, especially in the early phase (39).

Several limitations of our study should be acknowledged. First, our analyses were performed on bulk PBMC populations, which could not definitively distinguish whether observed transcriptional and methylation changes reflected true cellular reprogramming versus alterations in immune cell subset composition. Peripheral immune changes after RFA may have arisen partially from shifts in PBMC proportions rather than genuine transcriptional/epigenetic reprogramming at the single-cell level. We did not perform cell composition estimation or adjustment in this study, which could have confounded our signature scores. While we focused on T-cell differentiation gene sets that captured functional states beyond mere abundance, future studies using single-cell RNA sequencing and methylome profiling would be essential to validate whether these signatures represented intrinsic reprogramming within specific lineages. Second, the sample size was relatively small (n = 12), and despite longitudinal sampling over 14 months, larger cohorts were needed to validate these findings. Finally, this study primarily focused on peripheral immune changes; future work should integrate tumor tissue samples to comprehensively understand the interplay between RFA-induced systemic and local immune responses.

In conclusion, we reported the complex effect of the location of CpG site methylation on its corresponding gene expression. The methylation levels of CpG sites in the promoter region (≤1 kb) were mainly negatively correlated with transcription. RFA treatment in HCC patients activated multiple immune responses, especially T cell-related signaling pathways. The anti-tumor immune responses induced by RFA persisted for less than 9 months. Meanwhile, validated by public data, T-cell differentiation ability in HCC patients was a potential factor to prevent recurrence.

## Data Availability

The datasets presented in this article are not readily available due to privacy and ethical restrictions. The datasets are available upon reasonable request from the corresponding author.

## References

[1] Napoletano C Taurino F Biffoni M De Majo A Coscarella G Bellati F . RFA strongly modulates the immune system and anti-tumor immune responses in metastatic liver patients. Int J Oncol. (2008) 32:481–90. doi: 10.3892/ijo.32.2.481, PMID: 18202772

[2] Mizukoshi E Yamashita T Arai K Sunagozaka H Ueda T Arihara F . Enhancement of tumor-associated antigen-specific T cell responses by radiofrequency ablation of hepatocellular carcinoma. Hepatol (Baltimore Md). (2013) 57:1448–57. doi: 10.1002/hep.26153, PMID: 23174905

[3] Ali MY Grimm CF Ritter M Mohr L Allgaier HP Weth R . Activation of dendritic cells by local ablation of hepatocellular carcinoma. J hepatology. (2005) 43:817–22. doi: 10.1016/j.jhep.2005.04.016, PMID: 16087270

[4] Ji L Gu J Chen L Miao D . Changes of Th1/Th2 cytokines in patients with primary hepatocellular carcinoma after ultrasound-guided ablation. Int J Clin Exp pathology. (2017) 10:8715–20., PMID: 31966730 PMC6965388

[5] Erös de Bethlenfalva-Hora C Mertens JC Piguet AC Kettenbach J Schmitt J Terracciano L . Radiofrequency ablation suppresses distant tumour growth in a novel rat model of multifocal hepatocellular carcinoma. Clin Sci (London England: 1979). (2014) 126:243–52. doi: 10.1042/CS20130089, PMID: 23822114

[6] Nobuoka D Motomura Y Shirakawa H Yoshikawa T Kuronuma T Takahashi M . Radiofrequency ablation for hepatocellular carcinoma induces glypican-3 peptide-specific cytotoxic T lymphocytes. Int J Oncol. (2012) 40:63–70. doi: 10.3892/ijo.2011.1202, PMID: 21922136

[7] den Brok MH Sutmuller RP Nierkens S Bennink EJ Frielink C Toonen LW . Efficient loading of dendritic cells following cryo and radiofrequency ablation in combination with immune modulation induces anti-tumour immunity. Br J cancer. (2006) 95:896–905. doi: 10.1038/sj.bjc.6603341, PMID: 16953240 PMC2360548

[8] Gameiro SR Higgins JP Dreher MR Woods DL Reddy G Wood BJ . Combination therapy with local radiofrequency ablation and systemic vaccine enhances antitumor immunity and mediates local and distal tumor regression. PloS One. (2013) 8:e70417. doi: 10.1371/journal.pone.0070417, PMID: 23894654 PMC3722166

[9] Zeng X Liao G Li S Liu H Zhao X Li S . Eliminating METTL1-mediated accumulation of PMN-MDSCs prevents hepatocellular carcinoma recurrence after radiofrequency ablation. Hepatol (Baltimore Md). (2023) 77:1122–38. doi: 10.1002/hep.32585, PMID: 35598182

[10] Qin S Chen M Cheng AL Kaseb AO Kudo M Lee HC . Atezolizumab plus bevacizumab versus active surveillance in patients with resected or ablated high-risk hepatocellular carcinoma (IMbrave050): a randomised, open-label, multicentre, phase 3 trial. Lancet (London England). (2023) 402:1835–47. doi: 10.1016/S0140-6736(23)01796-8, PMID: 37871608

[11] Qiao W Wang Q Hu C Zhang Y Li J Sun Y . Interim efficacy and safety of PD-1 inhibitors in preventing recurrence of hepatocellular carcinoma after interventional therapy. Front Immunol. (2022) 13:1019772. doi: 10.3389/fimmu.2022.1019772, PMID: 36389724 PMC9650042

[12] den Brok MH Sutmuller RP van der Voort R Bennink EJ Figdor CG Ruers TJ . *In situ* tumor ablation creates an antigen source for the generation of antitumor immunity. Cancer Res. (2004) 64:4024–9. doi: 10.1158/0008-5472.CAN-03-3949, PMID: 15173017

[13] Wu TD Madireddi S de Almeida PE Banchereau R Chen Y-JJ Chitre AS . Peripheral T cell expansion predicts tumour infiltration and clinical response. Nature. (2020) 579:274–8. doi: 10.1038/s41586-020-2056-8, PMID: 32103181

[14] Zhao Y Yang T Ouyang Y Rao W Liu K Zheng J . Radiofrequency ablation plays double role in immunosuppression and activation of PBMCs in recurrent hepatocellular carcinoma. Front Immunol. (2024) 15:1339213. doi: 10.3389/fimmu.2024.1339213, PMID: 38348038 PMC10859425

[15] Chen S Zhou Y Chen Y Gu J . fastp: an ultra-fast all-in-one FASTQ preprocessor. Bioinf (Oxford England). (2018) 34:i884–i90. doi: 10.1093/bioinformatics/bty560, PMID: 30423086 PMC6129281

[16] Krueger F Andrews SR . Bismark: a flexible aligner and methylation caller for Bisulfite-Seq applications. Bioinf (Oxford England). (2011) 27:1571–2. doi: 10.1093/bioinformatics/btr167, PMID: 21493656 PMC3102221

[17] Andrews S . FastQC: a quality control tool for high throughput sequence data (2010). Available online at: http://www.bioinformatics.babraham.ac.uk/projects/fastqc/ (Accessed July 10, 2024).

[18] Patel RK Jain M . NGS QC Toolkit: a toolkit for quality control of next generation sequencing data. PloS One. (2012) 7:e30619. doi: 10.1371/journal.pone.0030619, PMID: 22312429 PMC3270013

[19] Sirén J Välimäki N Mäkinen V . Indexing graphs for path queries with applications in genome research. IEEE/ACM Trans Comput Biol Bioinf. (2014) 11:375–88. doi: 10.1109/TCBB.2013.2297101, PMID: 26355784

[20] Pertea M Pertea GM Antonescu CM Chang T-C Mendell JT Salzberg SL . StringTie enables improved reconstruction of a transcriptome from RNA-seq reads. Nat Biotechnol. (2015) 33:290–5. doi: 10.1038/nbt.3122, PMID: 25690850 PMC4643835

[21] Singh A Shannon CP Gautier B Rohart F Vacher M Tebbutt SJ . DIABLO: an integrative approach for identifying key molecular drivers from multi-omics assays. Bioinf (Oxford England). (2019) 35:3055–62. doi: 10.1093/bioinformatics/bty1054, PMID: 30657866 PMC6735831

[22] Ramos PS Zimmerman KD Haddad S Langefeld CD Medsger TA Jr. Feghali-Bostwick CA . Integrative analysis of DNA methylation in discordant twins unveils distinct architectures of systemic sclerosis subsets. Clin epigenetics. (2019) 11:58. doi: 10.1186/s13148-019-0652-y, PMID: 30947741 PMC6449959

[23] de Mendoza A Nguyen TV Ford E Poppe D Buckberry S Pflueger J . Large-scale manipulation of promoter DNA methylation reveals context-specific transcriptional responses and stability. Genome Biol. (2022) 23:163. doi: 10.1186/s13059-022-02728-5, PMID: 35883107 PMC9316731

[24] Miller RH Pollard CA Brogaard KR Olson AC Barney RC Lipshultz LI . Tissue-specific DNA methylation variability and its potential clinical value. Front Genet. (2023) 14:1125967. doi: 10.3389/fgene.2023.1125967, PMID: 37538359 PMC10394514

[25] Zhang Y Petropoulos S Liu J Cheishvili D Zhou R Dymov S . The signature of liver cancer in immune cells DNA methylation. Clin epigenetics. (2018) 10:8. doi: 10.1186/s13148-017-0436-1, PMID: 29375724 PMC5774119

[26] Li Y Fan Z Meng Y Liu S Zhan H . Blood-based DNA methylation signatures in cancer: A systematic review. Biochim Biophys Acta Mol basis disease. (2023) 1869:166583. doi: 10.1016/j.bbadis.2022.166583, PMID: 36270476

[27] Yang X Han H De Carvalho DD Lay FD Jones PA Liang G . Gene body methylation can alter gene expression and is a therapeutic target in cancer. Cancer Cell. (2014) 26:577–90. doi: 10.1016/j.ccr.2014.07.028, PMID: 25263941 PMC4224113

[28] Kitamura E Igarashi J Morohashi A Hida N Oinuma T Nemoto N . Analysis of tissue-specific differentially methylated regions (TDMs) in humans. Genomics. (2007) 89:326–37. doi: 10.1016/j.ygeno.2006.11.006, PMID: 17188838 PMC1847344

[29] Hansler J Wissniowski TT Schuppan D Witte A Bernatik T Hahn EG . Activation and dramatically increased cytolytic activity of tumor specific T lymphocytes after radio-frequency ablation in patients with hepatocellular carcinoma and colorectal liver metastases. World J gastroenterology. (2006) 12:3716–21. doi: 10.3748/wjg.v12.i23.3716, PMID: 16773688 PMC4087464

[30] Zhao Y Li K Sun J He N Zhao P Zang C . Genomic DNA methylation profiling indicates immune response following thermal ablation treatment for HBV-associated hepatocellular carcinoma. Oncol letters. (2020) 20:677–84. doi: 10.3892/ol.2020.11636, PMID: 32565992 PMC7285841

[31] Prokhnevska N Cardenas MA Valanparambil RM Sobierajska E Barwick BG Jansen C . CD8(+) T cell activation in cancer comprises an initial activation phase in lymph nodes followed by effector differentiation within the tumor. Immunity. (2023) 56:107–24.e5. doi: 10.1016/j.immuni.2022.12.002, PMID: 36580918 PMC10266440

[32] Connolly KA Kuchroo M Venkat A Khatun A Wang J William I . A reservoir of stem-like CD8(+) T cells in the tumor-draining lymph node preserves the ongoing antitumor immune response. Sci Immunol. (2021) 6:eabg7836. doi: 10.1126/sciimmunol.abg7836, PMID: 34597124 PMC8593910

[33] Krishna S Lowery FJ Copeland AR Bahadiroglu E Mukherjee R Jia L . Stem-like CD8 T cells mediate response of adoptive cell immunotherapy against human cancer. Sci (New York NY). (2020) 370:1328–34. doi: 10.1126/science.abb9847, PMID: 33303615 PMC8883579

[34] Siddiqui I Schaeuble K Chennupati V Fuertes Marraco SA Calderon-Copete S Pais Ferreira D . Intratumoral tcf1(+)PD-1(+)CD8(+) T cells with stem-like properties promote tumor control in response to vaccination and checkpoint blockade immunotherapy. Immunity. (2019) 50:195–211.e10. doi: 10.1016/j.immuni.2018.12.021, PMID: 30635237

[35] Sade-Feldman M Yizhak K Bjorgaard SL Ray JP de Boer CG Jenkins RW . Defining T cell states associated with response to checkpoint immunotherapy in melanoma. Cell. (2018) 175:998–1013.e20. doi: 10.1016/j.cell.2018.10.038, PMID: 30388456 PMC6641984

[36] Lindner AK Martowicz A Untergasser G Haybaeck J Compérat E Kocher F . CXCR3 expression is associated with advanced tumor stage and grade influencing survival after surgery of localised renal cell carcinoma. Cancers. (2023) 15. doi: 10.3390/cancers15041001, PMID: 36831346 PMC9954014

[37] Dromi SA Walsh MP Herby S Traughber B Xie J Sharma KV . Radiofrequency ablation induces antigen-presenting cell infiltration and amplification of weak tumor-induced immunity. Radiology. (2009) 251:58–66. doi: 10.1148/radiol.2511072175, PMID: 19251937 PMC2663580

[38] Rovere-Querini P Manfredi AA . Tumor destruction and in *situ* delivery of antigen presenting cells promote anti-neoplastic immune responses: implications for the immunotherapy of pancreatic cancer. JOP: J pancreas. (2004) 5:308–14., PMID: 15254366

[39] Ahn E Araki K Hashimoto M Li W Riley JL Cheung J . Role of PD-1 during effector CD8 T cell differentiation. Proc Natl Acad Sci United States America. (2018) 115:4749–54. doi: 10.1073/pnas.1718217115, PMID: 29654146 PMC5939075

