# Supplementary material for: Dynamics of peripheral immune signature identified by multi-omics and its impact on recurrence after radiofrequency ablation of hepatocellular carcinoma
Source: Front Immunol. 2026 Feb 20;17:1760329. doi: 10.3389/fimmu.2026.1760329 (PMC12962958; doi:10.3389/fimmu.2026.1760329)
Supplement: Supplementary file 2 [file DataSheet1.docx]

Supplementary Material

## Supplementary Figures


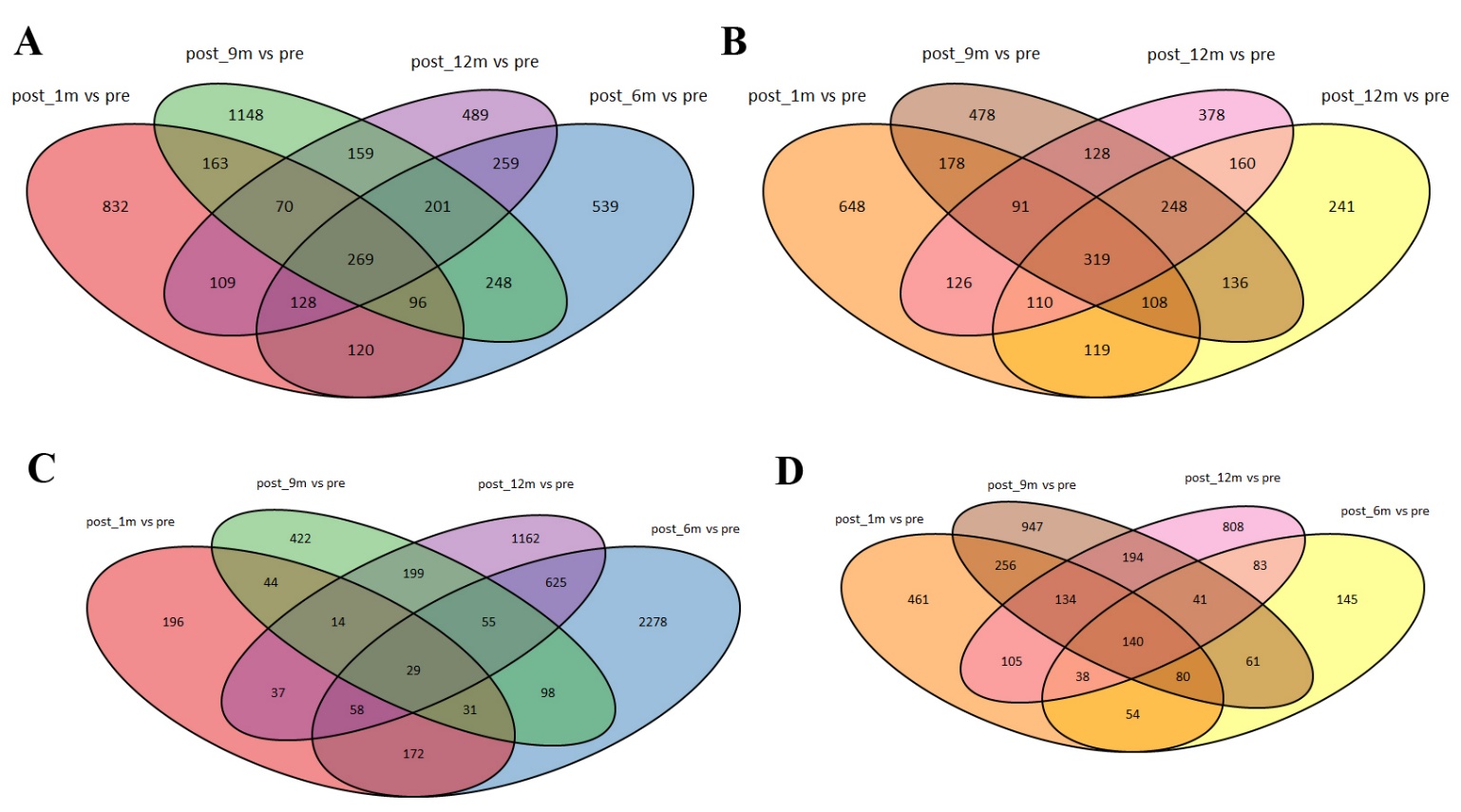


**Supplementary Figure 1.** Differential gene expression in each time period after RFA by Venn diagram analysis. (A) Number of differentially upregulated expression genes in non-recurrent patient. (B) Number of differentially downregulated expression genes in non-recurrent patient. (C) Number of differentially upregulated expression genes in recurrent patient. (D) Number of differentially downregulated expression genes in recurrent patient.


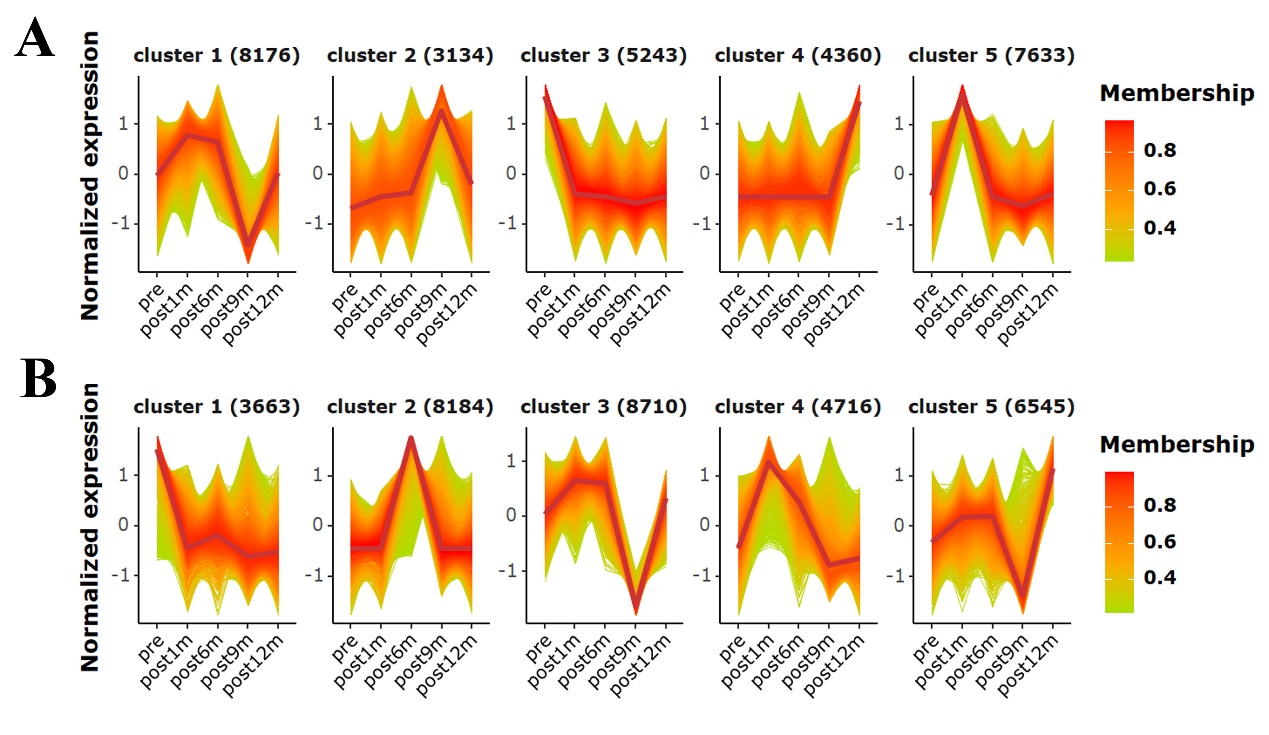


**Supplementary Figure 2.** Clustering analysis of the differentially expressed (DE) genes in non-recurrent patients (A) and recurrent patients (B). The DEGs were clustered into five sub-clusters. Number of genes in each cluster is shown at the top of each cluster.


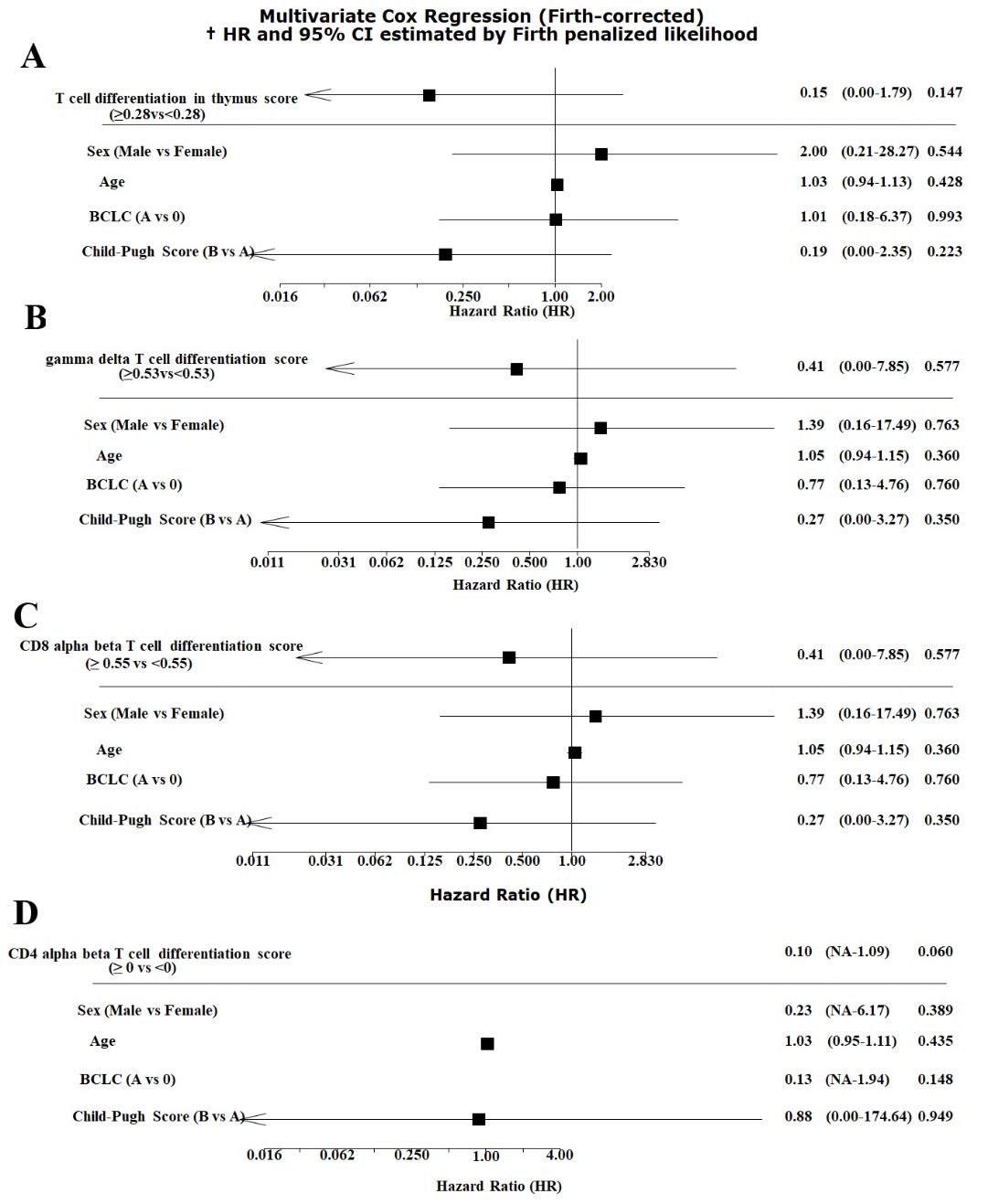


**Supplementary Figure 3.** **Multivariate cox regression (Firth-corrected) analyses of T cell differentiation signatures and clinical covariates in pre-ablation treatment samples (n=12). (A)** GOBP regulation of T cell differentiation in thymus; **(B)** GOBP regulation of gamma delta T cell differentiation; **(C)** GOBP positive regulation of CD8 positive alpha beta T cell differentiation; **(D)** GOBP positive regulation of CD4 positive alpha beta T cell differentiation. Error bars represent 95% CIs calculated by the Profile Likelihood method. HRs (95% CIs) and p-values are displayed on the right. The vertical red line indicates HR = 1 (no effect). † denoted HR estimated by Firth penalized likelihood due to complete separation. BCLC: Barcelona Clinic Liver Cancer Staging System. GOBP: gene ontology biological process.
